# Multi-valvular burden score and one-year mortality in ICU patients undergoing echocardiography: an exploratory cohort study

**DOI:** 10.1186/s44156-026-00139-x

**Published:** 2026-10-05

**Authors:** Hasan Burak Isleyen, Sevil Tugrul Yavuz, Sercan Bulut, Fatih Kizkapan, Cevahir Alioglu, Ali Arda Sozen, Mahsa Khanmohammadi

**Affiliations:** Department of Cardiology, Nisantasi University Faculty of Medicine, Istanbul, Turkey

**Keywords:** Valvular regurgitation, Multivalvular disease, Intensive care, Echocardiography, MIMIC-IV, Mortality, Cox regression

## Abstract

**Background:**

Multiple regurgitant lesions coexist in critically ill patients, yet cohort-level analyses typically represent each valve separately. We evaluated whether a Multi-Valvular Burden (MVB) score could summarize regurgitant involvement and whether its prognostic information differed from models retaining individual valve grades.

**Methods:**

This retrospective cohort used MIMIC-IV v3.1 linked with MIMIC-IV-ECHO. Adults with an ICU admission and complete ordinal grading of tricuspid, mitral, and aortic regurgitation on an index echocardiogram within 72 h of ICU admission were included. MVB was the arithmetic sum of TR, MR, and AR grades (0–12). Cox models assessed 1-year all-cause mortality. Additional analyses compared MVB with separate valve-grade models, tested a high-burden definition requiring MVB > = 4 and at least two involved valves, used missing-aware AS/MS evidence proxies, and examined LVEF with structured RV-function classification.

**Results:**

The cohort included 5,362 patients; 1,979 (36.9%) died within 1 year. Mortality increased from 24.6% at MVB 0 to 47.4% at MVB > = 4. After clinical adjustment, continuous MVB remained associated with mortality (HR 1.07 per point, 95% CI 1.05–1.09; *P* < 0.001), and MVB > = 4 was associated with higher mortality (HR 1.35, 95% CI 1.22–1.48; *P* < 0.001). The high-burden category predominantly reflected multivalve involvement: 2,206 of 2,257 patients with MVB > = 4 had at least two affected valves. A model with separate TR, MR, and AR grades showed similar discrimination to the MVB model (C-index 0.7585 vs. 0.7582). Incremental discrimination over the clinical model was small (C-index 0.7543 to 0.7582; delta 0.0039), although likelihood-ratio improvement was statistically significant (chi-square 50.61; *P* < 0.001). MVB remained associated with mortality in the missing-aware AS/MS sensitivity model (HR 1.07, 95% CI 1.05–1.09) and the LVEF/structured RV-function subset (HR 1.05, 95% CI 1.03–1.08).

**Conclusions:**

MVB is an exploratory summary marker rather than an outcome-derived or mechanistic weighting system. Its near-identical discrimination to a model retaining individual valve grades and very modest incremental discrimination suggest that it is best viewed as a compact descriptive marker of broader cardiovascular burden within systemic critical illness, not as a stand-alone clinical decision tool or evidence of a causal pathway to mortality.

**Clinical trial number:**

Not applicable.

**Trial registration:**

Not applicable.

**Supplementary Information:**

The online version contains supplementary material available at https://doi.org/10.1186/s44156-026-00139-x.

## Introduction

Valvular regurgitation is common in older and critically ill populations [1, 2]. Current valvular-heart-disease guidelines grade each valve separately because lesion severity, chamber adaptation, loading conditions, and treatment implications differ across the tricuspid, mitral, and aortic valves [3, 4]. The 2025 ESC/EACTS and ACC/AHA guidance also emphasize the complexity of multivalvular disease, in which management cannot be reduced to a single isolated lesion [3, 4].

For cohort-level description, however, a compact summary measure may provide a transparent way to represent the coexistence and cumulative severity of regurgitant lesions without requiring a separate parameter for each valve. We therefore constructed the MVB score as the simple arithmetic sum of ordinal TR, MR, and AR grades. The intended role of this score is descriptive: to provide a reproducible summary of regurgitant involvement for risk-adjusted cohort analyses and to test whether this compact representation retains information similar to that of the individual valve grades.

This simplification necessarily assumes equal ordinal contributions from physiologically heterogeneous lesions and does not imply that similarly summed values represent equivalent mechanisms. Severe isolated TR, mild regurgitation across three valves, and combined MR/AR may therefore have different biological meanings despite similar scores [5]. We evaluated MVB as an exploratory summary marker rather than a stand-alone prognostic instrument. The objectives were to quantify its association with 1-year mortality, compare MVB with models containing individual TR/MR/AR grades, test whether the high-burden threshold predominantly reflected multivalve involvement, and assess whether the association persisted after accounting for database-derived AS/MS evidence, LVEF, and structured RV function.

## Methods

### Study design and data source

A retrospective cohort study was performed using MIMIC-IV v3.1 and MIMIC-IV-ECHO, both available through PhysioNet [6–8]. The analysis followed STROBE and RECORD reporting principles [9, 10].

### Study population

Eligible patients were adults with an ICU stay and a clinically obtained transthoracic echocardiogram within 72 h of ICU admission. The index record required structured grading for tricuspid, mitral, and aortic regurgitation. One admission per patient was retained. The final cohort contained 5,362 patients, and the unit of analysis was the patient-level index ICU-echo episode.

### Exposure

TR, MR, and AR were mapped to ordinal grades from 0 to 4. MVB was the arithmetic sum of the three grades. This score is not outcome-derived and does not imply equal physiology across valves; it is a transparent summary of cumulative regurgitant severity. Primary analyses treated MVB continuously and as categories 0, 1, 2, 3, and > = 4. To assess whether the > = 4 threshold predominantly represented involvement of more than one valve, we additionally evaluated a high-burden definition requiring MVB > = 4 and regurgitation of any grade in at least two valves.

### Covariates

The clinical model included age, sex, SOFA, APS III, Charlson comorbidity index, myocardial infarction, congestive heart failure, stroke, chronic pulmonary disease, liver disease, diabetes, chronic kidney disease, malignancy, vasopressor use, mechanical ventilation, renal replacement therapy, creatinine, hemoglobin, and mean arterial pressure.

### Additional echocardiographic sensitivity variables

Structured measurements were obtained from the MIMIC-IV-ECHO structured_measurement table within +/-6 hours of the index echocardiogram. When multiple values of the same measurement type occurred within this window, the value temporally closest to the index echocardiogram was retained. The database-derived AS evidence proxy was positive when structured text indicated moderate or greater AS or low-flow AS, or when mean aortic-valve gradient was > = 20 mmHg, peak aortic-valve velocity was > = 3.0 m/s, or aortic-valve area was < = 1.5 cm2. Three internally discordant AS numeric records (two mean gradients > 200 mmHg accompanied by peak velocities of 1.3 m/s and one valve-area value of 0 cm2 accompanied by a peak velocity of 1.2 m/s, all without corroborating structured AS text) were excluded from proxy derivation. The database-derived MS evidence proxy was positive when structured text indicated moderate or greater MS, mean transmitral gradient was > = 5 mmHg, or an available mitral-valve-area measurement was < = 1.5 cm2. The pressure-half-time-related structured field represented mitral valve area in cm2; raw pressure half-time in milliseconds was not used. Indicators for unavailable AS and MS source data were included so that absence of recorded measurements was not treated as evidence of absent stenosis. Structured RV function was obtained from rv_function_extra with fallback to rv_function. Explicit RV dysfunction was defined by descriptions of hypokinesis/hypofunction or depressed function; ‘low normal’ was not classified as dysfunction. ‘RV not well seen’ and ‘cannot assess RV function’ were classified separately as indeterminate, and absent RV-function text was represented by a separate missingness indicator. TAPSE was available for descriptive analysis but was not used to construct the RV-function classification. Detailed source availability and numeric distributions are reported in Supplementary Table S3A.

### Outcome

The primary endpoint was 1-year all-cause mortality from the index echocardiogram. Follow-up was administratively censored at 365 days.

### Statistical analysis

Cox proportional hazards models estimated hazard ratios with 95% confidence intervals [11, 12]. The proportional hazards assumption was evaluated using Schoenfeld residuals. Because the MVB term showed a small but statistically significant departure from proportionality in the primary model, Cox estimates were interpreted as average associations over the 365-day follow-up rather than as strictly time-invariant effects. Restricted cubic spline analysis with three knots was used to assess non-linearity in the continuous MVB-mortality association. Model performance was compared across the clinical model, clinical plus MVB, clinical plus separate TR/MR/AR grades, a missing-aware model containing MVB plus AS/MS evidence proxies and source-availability indicators, and a model containing MVB, LVEF, and structured RV-function categories. For the LVEF/RV-function analysis, the clinical reference model was refit in the same complete-case subset. Incremental value was summarized using Harrell’s C-index with bootstrap confidence intervals, likelihood-ratio tests, continuous NRI and IDI, and VIF diagnostics [13, 14]. Because MVB is the exact sum of the three component valve grades, models containing MVB and all three component grades are structurally collinear and were interpreted only as penalized sensitivity analyses in the supplement. Statistical analyses were performed using Python 3.10 (Python Software Foundation, Wilmington, DE, USA) with pandas, NumPy, SciPy, statsmodels, lifelines, and scikit-learn packages.

## Results

### Cohort

**Fig. 1:**
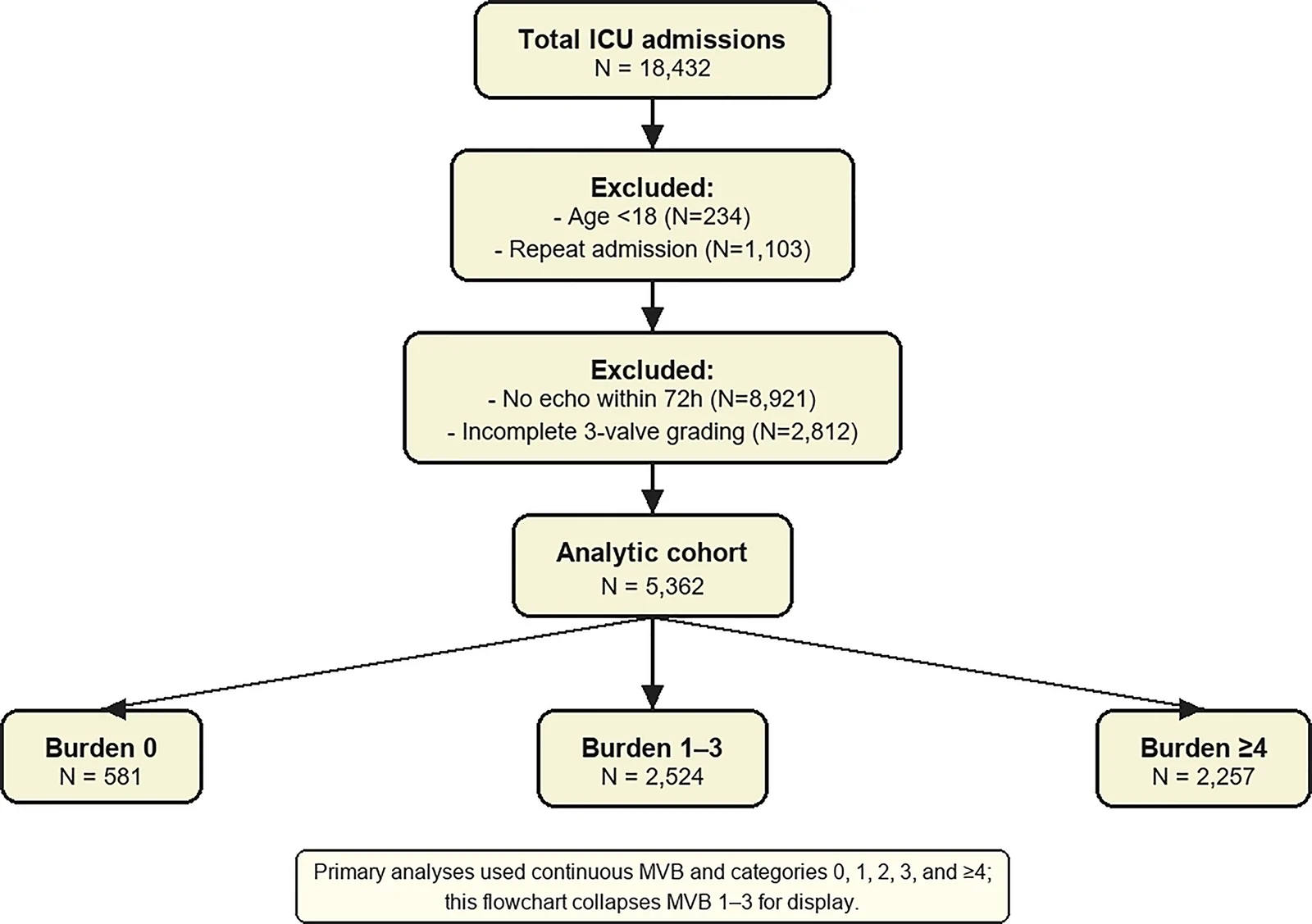
Cohort derivation flowchart. The source population consisted of adult ICU patients with an available index echocardiogram within 72 h of ICU admission. Patients without complete structured grading for tricuspid, mitral, and aortic regurgitation were excluded. The final analytic cohort included 5,362 patients. For display, MVB categories 1–3 were collapsed in the flowchart; primary analyses used continuous MVB and categories 0, 1, 2, 3, and ≥ 4

The final cohort included 5,362 patients, of whom 1,979 died within 1 year (Fig. 1). MVB scores ranged from 0 to 12; four patients had an MVB score of 12. The high-burden group (MVB > = 4) included 2,257 patients; 2,206 had at least two affected valves, while 51 reached the threshold from a single valve (Table 1).

**Table 1:** MVB categories, component valve involvement, and cardiac-function markers

MVB category	*N*	Mean MVB score	1-year deaths, *n* (%)	Any AR, *n* (%)	Any MR, *n* (%)	Any TR, *n* (%)	>=2 valves involved, *n* (%)	LVEF available, *n* (%)	Median LVEF, %	Explicit RV dysfunction, *n* (%)	Cleaned AS proxy positive, *n* (%)	MS proxy positive, *n* (%)
0	581	0.0	143 (24.6)	0 (0.0)	0 (0.0)	0 (0.0)	0 (0.0)	446 (76.8)	55 [50–65]	47 (8.1)	36 (6.2)	4 (0.7)
1	851	1.0	218 (25.6)	290 (34.1)	251 (29.5)	310 (36.4)	0 (0.0)	641 (75.3)	55 [45–60]	106 (12.5)	73 (8.6)	20 (2.4)
2	914	2.0	285 (31.2)	413 (45.2)	618 (67.6)	609 (66.6)	726 (79.4)	702 (76.8)	55 [40–60]	177 (19.4)	131 (14.3)	28 (3.1)
3	759	3.0	263 (34.7)	415 (54.7)	592 (78.0)	652 (85.9)	639 (84.2)	613 (80.8)	55 [40–60]	181 (23.8)	134 (17.7)	24 (3.2)
>=4	2257	5.8	1070 (47.4)	1495 (66.2)	2111 (93.5)	2131 (94.4)	2206 (97.7)	1809 (80.2)	50 [30–60]	870 (38.5)	510 (22.6)	116 (5.1)

AS and MS columns represent database-derived evidence proxies. RV dysfunction denotes explicit structured RV dysfunction; indeterminate and missing RV-function categories and source-availability details are reported in the Supplementary Material

### Primary association

**Fig. 2:**
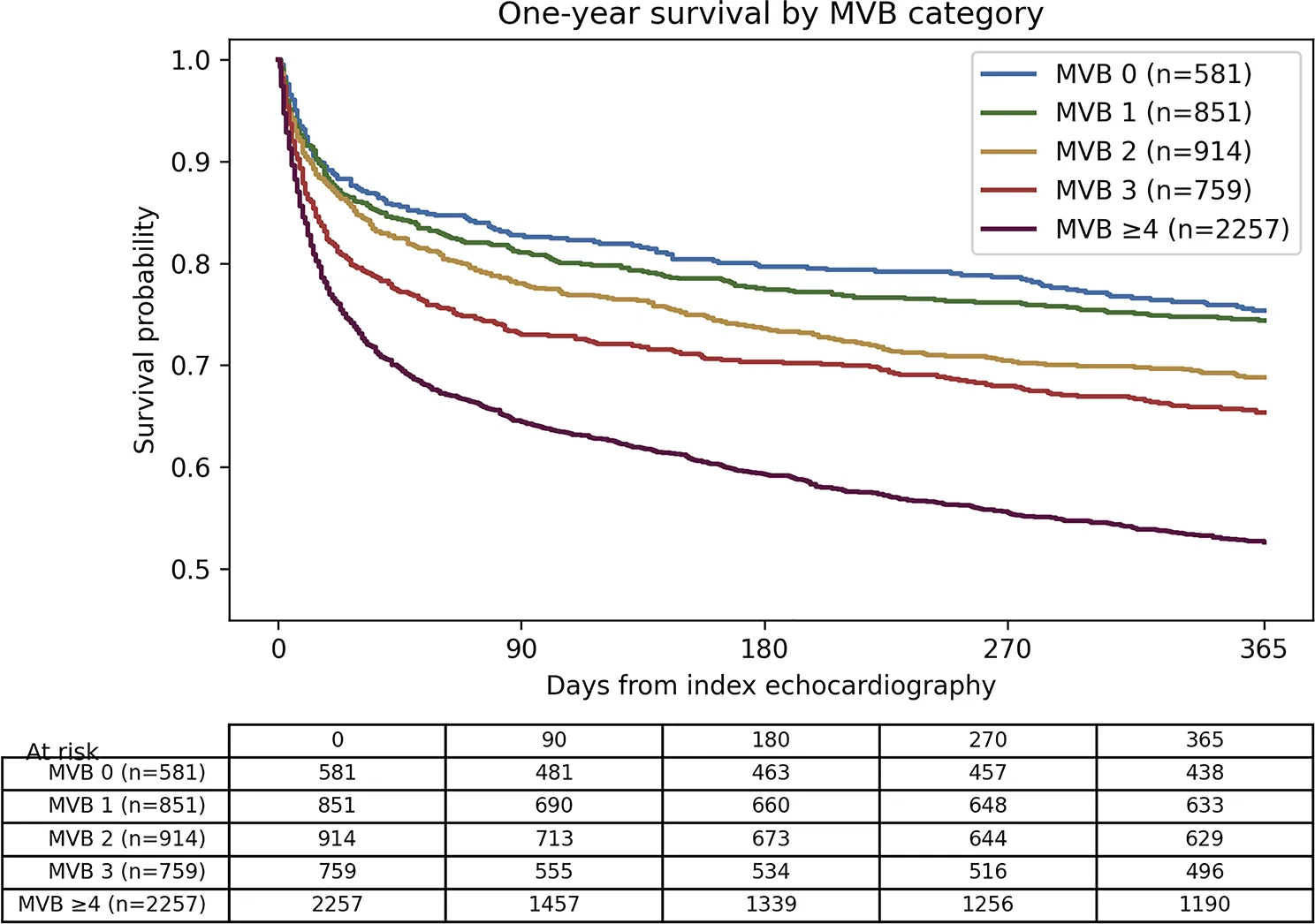
Kaplan–Meier survival curves by MVB category. Survival was estimated from the index echocardiogram to 365 days and stratified by MVB categories 0, 1, 2, 3, and ≥ 4. The curves are intended for descriptive comparison of unadjusted survival patterns

Unadjusted survival patterns across MVB categories are shown in Fig. 2. In the fully adjusted model, continuous MVB was associated with 1-year mortality (HR 1.07 per point, 95% CI 1.05–1.09; *P* < 0.001). Adjusted Cox models included 5,320 patients; 42 patients from the full cohort were excluded from adjusted modeling because one or more clinical covariates were missing. MVB > = 4 was also associated with mortality compared with lower burden (HR 1.35, 95% CI 1.22–1.48; *P* < 0.001). When the high-burden definition required at least two affected valves, the association was nearly unchanged (HR 1.34, 95% CI 1.22–1.47; *P* < 0.001). Schoenfeld residual testing showed a small departure from proportionality for continuous MVB in the primary model (*P* = 0.032); therefore, the hazard ratios are reported as average associations across follow-up. Restricted cubic spline analysis did not support material non-linearity in the MVB-mortality association (Pnon-linearity = 0.43).

### Individual component comparison

In a model including the three valve grades separately, TR (HR 1.09 per grade, 95% CI 1.05–1.14; *P* < 0.001) and MR (HR 1.06 per grade, 95% CI 1.02–1.11; *P* = 0.002) were independently associated with mortality, whereas AR was weaker and not statistically significant (HR 1.05, 95% CI 0.99–1.10; *P* = 0.099).

**Table 2:** Model performance and incremental value

Model	*N*	Events	Harrell C-index	C-index 95% CI	LR chi-square vs. clinical	LR df	LR *P* value
Clinical model	5320	1969	0.7543	0.7428–0.7616	Reference	Not applicable	Not applicable
Clinical + continuous MVB	5320	1969	0.7582	0.7489–0.7712	50.61	1	< 0.001
Clinical + separate TR/MR/AR grades	5320	1969	0.7585	0.7493–0.7703	52.44	3	< 0.001
Clinical + MVB + AS/MS evidence proxies + availability	5320	1969	0.7584	0.7512–0.7712	56.84	5	< 0.001
Clinical model (LVEF/RV subset)	4180	1546	0.7518	0.7398–0.7647	Reference	Not applicable	Not applicable
Clinical + MVB + LVEF/structured RV function	4180	1546	0.7597	0.7527–0.7714	89.00	5	< 0.001

The likelihood-ratio test for the LVEF/RV-function sensitivity model used the clinical model refit in the same 4,180-patient complete-case subset

### Incremental performance

Clinical model discrimination was 0.7543. Adding continuous MVB increased the C-index to 0.7582, a small absolute gain of 0.0039, despite a statistically significant likelihood-ratio improvement (chi-square 50.61; *P* < 0.001). Exploratory reclassification metrics are provided in Supplementary Table S5; no clinical risk-threshold reclassification was prespecified.

### Sensitivity analyses

In the missing-aware AS/MS model, cleaned AS proxy positivity was present in 884 patients and MS proxy positivity in 192 patients; all AS source data were unavailable in 296 patients and all MS source data in 4,160. With source-unavailability indicators included, MVB remained associated with mortality (HR 1.07 per point, 95% CI 1.05–1.09; *P* < 0.001; C-index 0.7584, 95% CI 0.7512–0.7712). The cleaned AS proxy was not independently associated with mortality (HR 1.08, 95% CI 0.96–1.22; *P* = 0.180), whereas AS source unavailability was associated with mortality (HR 1.25, 95% CI 1.03–1.50; *P* = 0.022); neither the MS proxy nor MS source unavailability was associated with mortality. LVEF was available in 4,211 patients. After complete-case restriction for the prespecified clinical covariates, the LVEF/RV-function sensitivity analysis included 4,180 patients and 1,546 deaths. MVB remained associated with mortality (HR 1.05 per point, 95% CI 1.03–1.08; *P* < 0.001), while lower LVEF (HR 0.99 per 1%, 95% CI 0.99-1.00; *P* = 0.008) and explicitly documented RV dysfunction (HR 1.40, 95% CI 1.23–1.58; *P* < 0.001) were also associated with mortality. The C-index was 0.7597 (95% CI 0.7527–0.7714), compared with 0.7518 (95% CI 0.7398–0.7647) for the clinical model refit in the same subset. Global proportional-hazards tests remained significant in both revised sensitivity models; the MVB term showed a small departure in the AS/MS model (*P* = 0.036) and did not cross the conventional threshold in the LVEF/RV model (*P* = 0.056). BNP/NT-proBNP was available for only 548 patients and remained an exploratory available-case analysis. Primary and sensitivity model estimates are summarized in Table 3. Revised sensitivity-model proportional-hazards diagnostics are provided in Supplementary Table S9A, and adjusted sensitivity estimates are summarized in Supplementary Figure S1.

**Table 3:** Primary, component-level, and sensitivity analyses

Model	*N*	Events	Term	HR (95% CI)	*P* value
Clinical + continuous MVB	5320	1969	MVB, per point	1.07 (1.05–1.09)	< 0.001
Clinical + MVB > = 4	5320	1969	MVB > = 4	1.35 (1.22–1.48)	< 0.001
Clinical + separate TR/MR/AR grades	5320	1969	TR grade, per point	1.09 (1.05–1.14)	< 0.001
Clinical + separate TR/MR/AR grades	5320	1969	MR grade, per point	1.06 (1.02–1.11)	0.002
Clinical + separate TR/MR/AR grades	5320	1969	AR grade, per point	1.05 (0.99–1.10)	0.099
Clinical + MVB > = 4 and > = 2 valves	5320	1969	MVB > = 4 and > = 2 valves	1.34 (1.22–1.47)	< 0.001
Clinical + MVB + AS/MS evidence proxies + availability	5320	1969	MVB, per point	1.07 (1.05–1.09)	< 0.001
Clinical + MVB + AS/MS evidence proxies + availability	5320	1969	Cleaned AS proxy positive	1.08 (0.96–1.22)	0.180
Clinical + MVB + AS/MS evidence proxies + availability	5320	1969	AS source unavailable	1.25 (1.03–1.50)	0.022
Clinical + MVB + AS/MS evidence proxies + availability	5320	1969	MS proxy positive	1.00 (0.78–1.27)	0.973
Clinical + MVB + AS/MS evidence proxies + availability	5320	1969	MS source unavailable	1.00 (0.89–1.11)	0.944
Clinical + MVB + LVEF/structured RV function	4180	1546	MVB, per point	1.05 (1.03–1.08)	< 0.001
Clinical + MVB + LVEF/structured RV function	4180	1546	LVEF, per 1%	0.99 (0.99-1.00)	0.008
Clinical + MVB + LVEF/structured RV function	4180	1546	Explicit RV dysfunction	1.40 (1.23–1.58)	< 0.001
Clinical + MVB + LVEF/structured RV function	4180	1546	RV function indeterminate	0.96 (0.67–1.36)	0.807
Clinical + MVB + LVEF/structured RV function	4180	1546	RV-function text unavailable	1.16 (0.84–1.62)	0.370
Clinical + MVB + BNP/NT-proBNP available-case	546	242	MVB, per point	1.04 (0.99–1.10)	0.122
Clinical + MVB + BNP/NT-proBNP available-case	546	242	Log BNP/NT-proBNP	1.10 (0.98–1.23)	0.096

All models were adjusted for the clinical covariate set described in the Methods. The AS/MS model additionally included source-unavailability indicators. The LVEF/RV model used explicit structured RV dysfunction with separate indeterminate and unavailable categories. MVB indicates Multi-Valvular Burden; TR, tricuspid regurgitation; MR, mitral regurgitation; AR, aortic regurgitation; AS, aortic stenosis; MS, mitral stenosis; LVEF, left ventricular ejection fraction; RV, right ventricular

### Subgroups

**Fig. 3:**
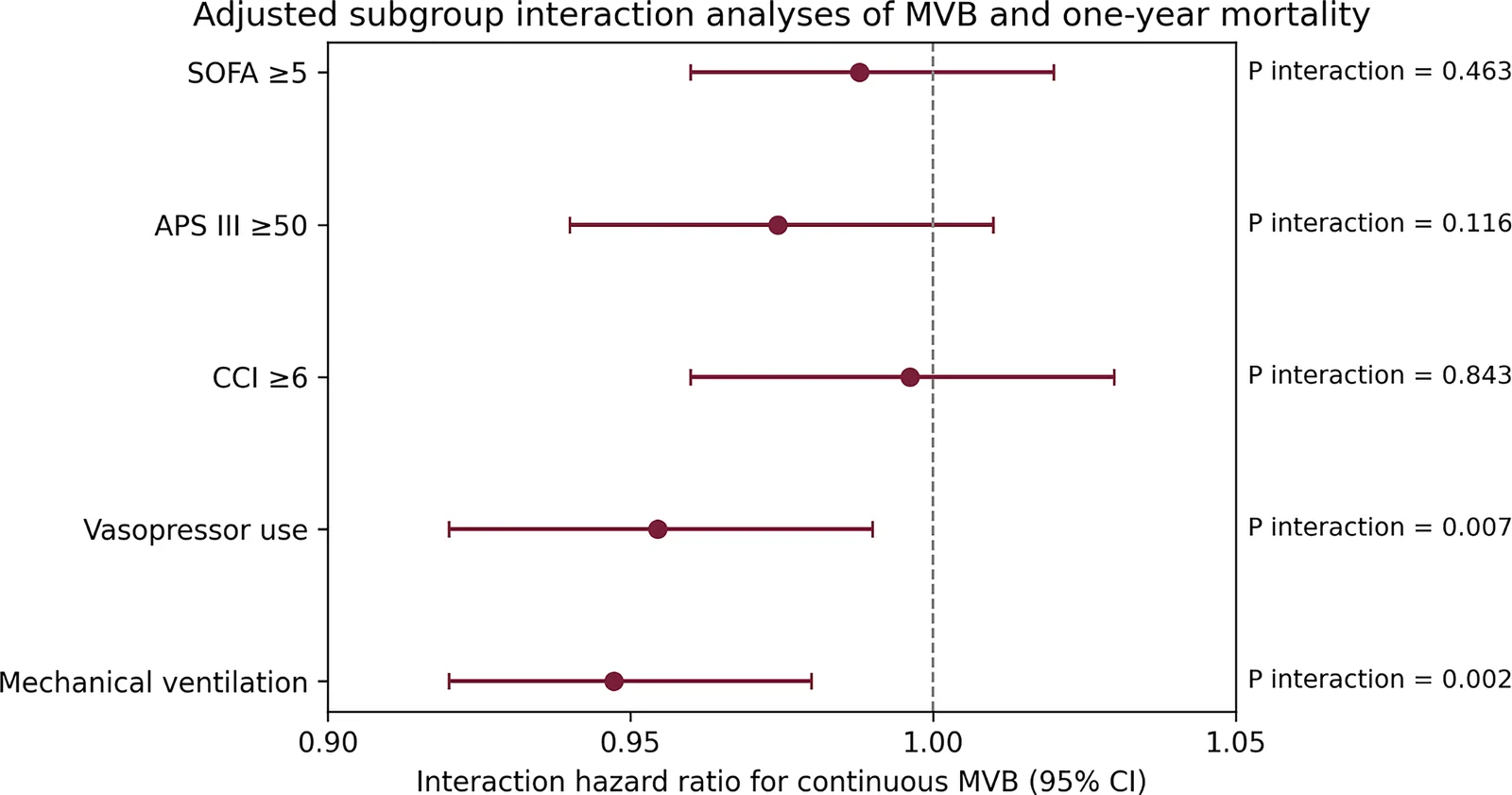
Adjusted subgroup interaction analyses for the association between continuous MVB and one-year mortality. Interaction terms were estimated in clinically adjusted Cox models using the covariate set described in the Methods. Interaction hazard ratios below 1 indicate attenuation of the MVB association in the specified subgroup. Interaction P values are shown on the plot

**Fig. 4:**
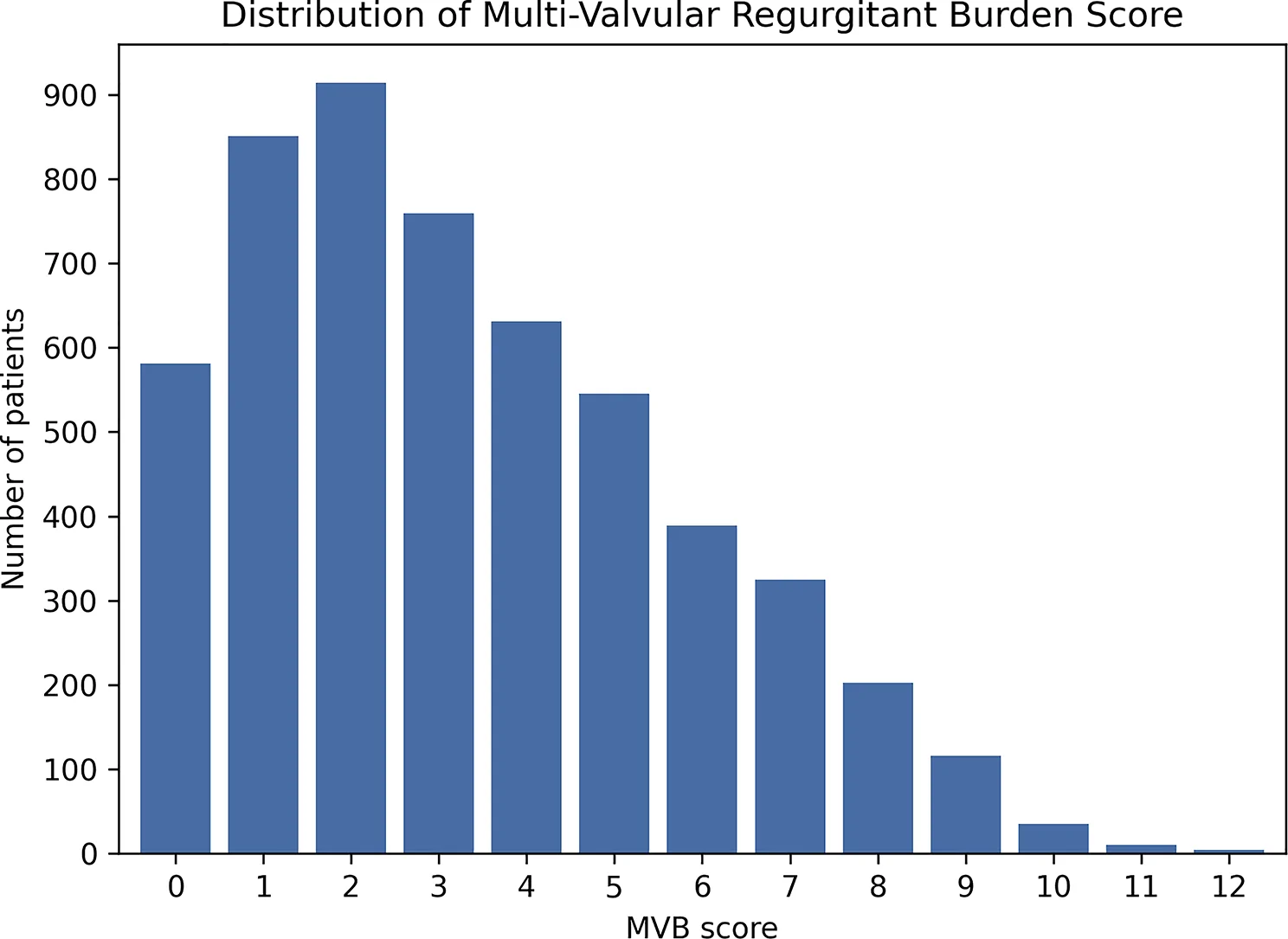
Distribution of MVB score in the analytic cohort. Bars show the number of patients across the full MVB range from 0 to 12. The distribution supports use of MVB as both a continuous exposure and a categorized descriptive marker

Adjusted subgroup interaction analyses are shown in Fig. 3. Adjusted interaction testing showed no evidence of interaction for SOFA > = 5, APS III > = 50, or CCI > = 6. The MVB association was attenuated among patients receiving vasopressors and mechanical ventilation, with statistically significant interaction terms (interaction *P* = 0.007 and 0.002, respectively). These findings suggest effect-size modification by acute organ-support status rather than uniform behavior across all ICU strata. The full MVB score distribution is shown in Fig. 4.

## Discussion

MVB was consistently associated with 1-year mortality, but the score should be understood as a simple arithmetic summary rather than an outcome-weighted or mechanistic score. Separate TR/MR/AR modeling showed that TR and MR contributed more clearly than AR, yet the model retaining all three valve grades had virtually identical discrimination to the MVB model (C-index 0.7585 vs. 0.7582).

The high-burden threshold had a distributional basis in this cohort. Although a patient could theoretically reach MVB > = 4 from a single severe lesion, only 51 of 2,257 high-burden patients did so. The threshold therefore mostly identified patients with multiple involved valves. This supports the multivalvular description of the categorical analysis but does not validate the threshold as a clinical decision point.

The near-identical discrimination of the MVB model and the model containing TR, MR, and AR grades separately may be informative rather than simply representing limited incremental performance. Within this selected critically ill cohort, much of the prognostic information carried by the individual regurgitant lesions could be summarized by their overall burden, with little additional discrimination from retaining valve identity. This does not imply that TR, MR, and AR are physiologically equivalent. Rather, higher MVB may act as a simple surrogate for a broader cardiovascular burden encompassing adverse chamber remodeling, ventricular dysfunction, abnormal loading conditions, and cardiovascular frailty.

This interpretation must also account for the use of all-cause mortality as the primary endpoint. Death in critically ill patients may result from cardiovascular causes but also from sepsis, respiratory failure, malignancy, neurological complications, and multiorgan dysfunction. The present data therefore cannot determine whether the association between MVB and mortality is mediated by a cardiovascular mechanism or whether higher MVB primarily identifies patients with greater overall illness severity and complexity. Accordingly, MVB is better interpreted as a compact descriptive marker of broader cardiovascular burden within systemic critical illness than as evidence that cumulative regurgitation itself causes mortality.

Available echocardiographic sensitivity analyses partly addressed residual cardiac confounding. The MVB association was stable after explicit modeling of AS/MS proxy positivity and source-data availability and after LVEF and structured RV-function classification. The association of AS source unavailability with mortality further illustrates that measurement availability may encode care processes or patient complexity and should not be given a biological interpretation. Quantitative TAPSE was available in only 1,273 patients and was not used to define RV dysfunction. The score also cannot distinguish organic from functional regurgitation, chamber-remodeling patterns, pulmonary-hypertension mechanisms, or dominant-lesion physiology, all of which remain central in guideline-based and multimodality valve assessment [15, 16].

### Limitations

This was a single-center retrospective database study of patients selected for clinically indicated echocardiography. Regurgitation grades were structured clinical measurements rather than blinded core-lab adjudications. MVB assigns equal ordinal weight to TR, MR, and AR despite biological heterogeneity. Several Cox-model terms showed departures from the proportional hazards assumption; the MVB term showed a small departure in the primary model and the revised AS/MS sensitivity model. Hazard ratios should therefore be interpreted as average associations over follow-up rather than strictly constant effects. AS/MS evidence was derived from incompletely available structured measurements, particularly for MS; source-unavailability indicators reduced but cannot eliminate measurement-selection bias. LVEF and quantitative RV measures such as TAPSE were available only in subsets. Because the primary endpoint was all-cause mortality, cause-specific cardiovascular death could not be distinguished from deaths related to systemic critical illness; consequently, the analysis cannot establish whether the observed association reflects a cardiovascular pathway or broader patient complexity. Residual confounding, treatment indication, transient hemodynamic effects, and selection associated with obtaining echocardiography cannot be excluded.

## Conclusions

In ICU patients undergoing echocardiography, an arithmetic MVB score was associated with 1-year all-cause mortality after adjustment for clinical severity. Its incremental discrimination was very small and virtually identical to a model using individual valve grades. These findings support interpreting MVB as an exploratory descriptive marker of broader cardiovascular burden within systemic critical illness, rather than as a distinct multivalvular mechanism, a clinical decision tool, or evidence that cumulative regurgitation causally contributes to mortality.

## Supplementary Information

Below is the link to the electronic supplementary material.

Supplementary Material 1

Supplementary Material 2

## Data Availability

The data that support the findings of this study are derived from the Medical Information Mart for Intensive Care IV (MIMIC-IV, version 3.1) database, which is publicly available through PhysioNet (https://doi.org/10.13026/kpb9-mt58). Access to the raw database requires completion of the required training and signing of the applicable Data Use Agreement through PhysioNet. The analytic dataset generated during the study is available from the corresponding author upon reasonable request, subject to the PhysioNet credentialed-access policy.
