# Supplementary material for: Multi-valvular burden score and one-year mortality in ICU patients undergoing echocardiography: an exploratory cohort study
Source: Echo Res Pract. 2026 Oct 5;13:36. doi: 10.1186/s44156-026-00139-x (PMC13637370; doi:10.1186/s44156-026-00139-x)
Supplement: Supplementary file 1 [file 44156_2026_139_MOESM1_ESM.docx]

# Supplementary Material

Multi-Valvular Burden Score and One-Year Mortality in ICU Patients Undergoing Echocardiography: An Exploratory Cohort Study

This supplement contains model comparisons, echocardiographic proxy definitions and source-data availability, full coefficients, collinearity diagnostics, subgroup interaction tests, proportional-hazards diagnostics, and sensitivity analyses.

**Supplementary Table S1. MVB score distribution**

| MVB score | N | 1-year deaths, n (%) | Mean number of involved valves | Patients with >=2 valves involved, n (%) |
| --- | --- | --- | --- | --- |
| 0 | 581 | 143 (24.6%) | 0.0 | 0 (0.0%) |
| 1 | 851 | 218 (25.6%) | 1.0 | 0 (0.0%) |
| 2 | 914 | 285 (31.2%) | 1.8 | 726 (79.4%) |
| 3 | 759 | 263 (34.7%) | 2.2 | 639 (84.2%) |
| 4 | 631 | 244 (38.7%) | 2.2 | 580 (91.9%) |
| 5 | 545 | 259 (47.5%) | 2.5 | 545 (100.0%) |
| 6 | 389 | 188 (48.3%) | 2.6 | 389 (100.0%) |
| 7 | 325 | 183 (56.3%) | 2.7 | 325 (100.0%) |
| 8 | 202 | 108 (53.5%) | 2.7 | 202 (100.0%) |
| 9 | 116 | 61 (52.6%) | 3.0 | 116 (100.0%) |
| 10 | 35 | 17 (48.6%) | 3.0 | 35 (100.0%) |
| 11 | 10 | 7 (70.0%) | 3.0 | 10 (100.0%) |
| 12 | 4 | 3 (75.0%) | 3.0 | 4 (100.0%) |

**Supplementary Table S2. MVB category and component-valve table**

| MVB category | N | Mean MVB score | 1-year deaths, n (%) | Any AR, n (%) | Any MR, n (%) | Any TR, n (%) | >=2 valves involved, n (%) | LVEF available, n (%) | Median LVEF, % | Explicit RV dysfunction, n (%) | Cleaned AS proxy positive, n (%) | MS proxy positive, n (%) |
| --- | --- | --- | --- | --- | --- | --- | --- | --- | --- | --- | --- | --- |
| 0 | 581 | 0.0 | 143 (24.6) | 0 (0.0) | 0 (0.0) | 0 (0.0) | 0 (0.0) | 446 (76.8) | 55 [50-65] | 47 (8.1) | 36 (6.2) | 4 (0.7) |
| 1 | 851 | 1.0 | 218 (25.6) | 290 (34.1) | 251 (29.5) | 310 (36.4) | 0 (0.0) | 641 (75.3) | 55 [45-60] | 106 (12.5) | 73 (8.6) | 20 (2.4) |
| 2 | 914 | 2.0 | 285 (31.2) | 413 (45.2) | 618 (67.6) | 609 (66.6) | 726 (79.4) | 702 (76.8) | 55 [40-60] | 177 (19.4) | 131 (14.3) | 28 (3.1) |
| 3 | 759 | 3.0 | 263 (34.7) | 415 (54.7) | 592 (78.0) | 652 (85.9) | 639 (84.2) | 613 (80.8) | 55 [40-60] | 181 (23.8) | 134 (17.7) | 24 (3.2) |
| >=4 | 2257 | 5.8 | 1070 (47.4) | 1495 (66.2) | 2111 (93.5) | 2131 (94.4) | 2206 (97.7) | 1809 (80.2) | 50 [30-60] | 870 (38.5) | 510 (22.6) | 116 (5.1) |

Note: Explicit RV dysfunction excludes low-normal, indeterminate, and unavailable RV-function assessments. The cleaned AS proxy excludes three internally discordant numeric records identified during the revision-stage data-quality audit. Proxy definitions and source-data availability are detailed in Supplementary Table S3A.

**Supplementary Table S3. Primary and sensitivity model terms**

| Model | N | Events | Term | HR (95% CI) | P value |
| --- | --- | --- | --- | --- | --- |
| Clinical + continuous MVB | 5320 | 1969 | MVB, per point | 1.07 (1.05-1.09) | <0.001 |
| Clinical + MVB >=4 | 5320 | 1969 | MVB >=4 | 1.35 (1.22-1.48) | <0.001 |
| Clinical + separate TR/MR/AR grades | 5320 | 1969 | TR grade, per point | 1.09 (1.05-1.14) | <0.001 |
| Clinical + separate TR/MR/AR grades | 5320 | 1969 | MR grade, per point | 1.06 (1.02-1.11) | 0.002 |
| Clinical + separate TR/MR/AR grades | 5320 | 1969 | AR grade, per point | 1.05 (0.99-1.10) | 0.099 |
| Clinical + MVB >=4 and >=2 valves | 5320 | 1969 | MVB >=4 and >=2 valves | 1.34 (1.22-1.47) | <0.001 |
| Clinical + MVB + AS/MS evidence proxies + availability | 5320 | 1969 | MVB, per point | 1.07 (1.05-1.09) | <0.001 |
| Clinical + MVB + AS/MS evidence proxies + availability | 5320 | 1969 | Cleaned AS proxy positive | 1.08 (0.96-1.22) | 0.180 |
| Clinical + MVB + AS/MS evidence proxies + availability | 5320 | 1969 | AS source unavailable | 1.25 (1.03-1.50) | 0.022 |
| Clinical + MVB + AS/MS evidence proxies + availability | 5320 | 1969 | MS proxy positive | 1.00 (0.78-1.27) | 0.973 |
| Clinical + MVB + AS/MS evidence proxies + availability | 5320 | 1969 | MS source unavailable | 1.00 (0.89-1.11) | 0.944 |
| Clinical + MVB + LVEF/structured RV function | 4180 | 1546 | MVB, per point | 1.05 (1.03-1.08) | <0.001 |
| Clinical + MVB + LVEF/structured RV function | 4180 | 1546 | LVEF, per 1% | 0.99 (0.99-1.00) | 0.008 |
| Clinical + MVB + LVEF/structured RV function | 4180 | 1546 | Explicit RV dysfunction | 1.40 (1.23-1.58) | <0.001 |
| Clinical + MVB + LVEF/structured RV function | 4180 | 1546 | RV function indeterminate | 0.96 (0.67-1.36) | 0.807 |
| Clinical + MVB + LVEF/structured RV function | 4180 | 1546 | RV-function text unavailable | 1.16 (0.84-1.62) | 0.370 |
| Clinical + MVB + BNP/NT-proBNP available-case | 546 | 242 | MVB, per point | 1.04 (0.99-1.10) | 0.122 |
| Clinical + MVB + BNP/NT-proBNP available-case | 546 | 242 | Log BNP/NT-proBNP | 1.10 (0.98-1.23) | 0.096 |

Supplementary Table S3A. Definitions, availability, and distributions of additional echocardiographic measurements

| Variable | Role / definition | Available N | Median [IQR] or n (%) | Mean +/- SD | Range |
| --- | --- | --- | --- | --- | --- |
| AV mean gradient | AS proxy component; criterion >=20 mmHg | 1538 | 15.0 [10.0-28.8] mmHg | 22.0 +/- 29.0 mmHg | 1-785 mmHg* |
| AV peak velocity | AS proxy component; criterion >=3.0 m/s | 5030 | 1.6 [1.3-2.1] m/s | 1.87 +/- 0.90 m/s | 0.5-6.2 m/s |
| AV area | AS proxy component; criterion <=1.5 cm2 | 1146 | 1.25 [0.9-1.7] cm2 | 1.38 +/- 0.67 cm2 | 0-4.7 cm2* |
| Any AS source available | Any AS text or proxy numeric component available | 5066 | 5066 (94.5%) | Not applicable | Not applicable |
| Cleaned AS proxy positive | Database-derived AS evidence proxy after revision-stage cleaning | 5362 | 884 (16.5%) | Not applicable | Not applicable |
| All AS sources unavailable | Separate missingness indicator in revised AS/MS model | 5362 | 296 (5.5%) | Not applicable | Not applicable |
| MV mean gradient | MS proxy component; criterion >=5 mmHg | 422 | 4 [3-6] mmHg | 5.08 +/- 3.23 mmHg | 1-19 mmHg |
| MVA, pressure-half-time-derived field | MS proxy component; structured field represents MVA in cm2; criterion <=1.5 cm2 | 100 | 2.75 [2.1-3.9] cm2 | 2.97 +/- 1.27 cm2 | 0.6-6.3 cm2 |
| MVA, planimetry | MS proxy component; criterion <=1.5 cm2 | 3 | 1.0 [1.0-1.25] cm2 | 1.17 +/- 0.29 cm2 | 1.0-1.5 cm2 |
| Generic MVA field | MS proxy component in code; no nonmissing values | 0 | Not available | Not available | Not available |
| Any MS source available | Any MS text or proxy numeric component available | 1202 | 1202 (22.4%) | Not applicable | Not applicable |
| MS proxy positive | Database-derived MS evidence proxy | 5362 | 192 (3.6%) | Not applicable | Not applicable |
| All MS sources unavailable | Separate missingness indicator in revised AS/MS model | 5362 | 4160 (77.6%) | Not applicable | Not applicable |
| Structured RV-function text | Source for structured RV classification | 5252 | 5252 (97.9%) | Not applicable | Not applicable |
| Explicit RV dysfunction | Hypokinesis/hypofunction or depressed function; low-normal excluded | 5362 | 1381 (25.8%) | Not applicable | Not applicable |
| RV function indeterminate | RV not well seen or function cannot be assessed | 5362 | 109 (2.0%) | Not applicable | Not applicable |
| RV-function text unavailable | Separate missingness category | 5362 | 110 (2.1%) | Not applicable | Not applicable |
| TAPSE | Descriptive quantitative RV measure; not used in RV-function classification | 1273 | 1.9 [1.6-2.2] cm | 1.92 +/- 0.53 cm | 0.4-3.9 cm |

*Original structured measurements included two internally discordant AV mean-gradient values >200 mmHg with peak velocity 1.3 m/s and one AV-area value of 0 cm2 with peak velocity 1.2 m/s, all without corroborating structured AS text. These three numeric entries were excluded from cleaned AS proxy derivation. Raw pressure half-time in milliseconds was not used; the pressure-half-time-related field represented mitral valve area in cm2.

**Supplementary Table S4. Model performance comparison**

| Model | N | Events | Harrell C-index | C-index 95% CI | LR chi-square vs clinical | LR df | LR P value |
| --- | --- | --- | --- | --- | --- | --- | --- |
| Clinical model | 5320 | 1969 | 0.7543 | 0.7428-0.7616 | Reference | Not applicable | Not applicable |
| Clinical + continuous MVB | 5320 | 1969 | 0.7582 | 0.7489-0.7712 | 50.61 | 1 | <0.001 |
| Clinical + separate TR/MR/AR grades | 5320 | 1969 | 0.7585 | 0.7493-0.7703 | 52.44 | 3 | <0.001 |
| Clinical + MVB + AS/MS evidence proxies + availability | 5320 | 1969 | 0.7584 | 0.7512-0.7712 | 56.84 | 5 | <0.001 |
| Clinical model (LVEF/RV subset) | 4180 | 1546 | 0.7518 | 0.7398-0.7647 | Reference | Not applicable | Not applicable |
| Clinical + MVB + LVEF/structured RV function | 4180 | 1546 | 0.7597 | 0.7527-0.7714 | 89.00 | 5 | <0.001 |

Note: The likelihood-ratio test for the LVEF/RV-function model used the clinical model refit in the same 4,180-patient complete-case subset.

**Supplementary Table S5. Continuous NRI and IDI**

| Comparison | Continuous NRI | Event component | Nonevent component | IDI |
| --- | --- | --- | --- | --- |
| Clinical model + continuous MVB vs clinical model | 0.200 | -0.005 | 0.204 | 0.007 |

Note: Continuous NRI and IDI were reported descriptively to supplement the likelihood-ratio and C-index analyses; no clinical risk-threshold reclassification was prespecified.

**Supplementary Table S6. VIF diagnostics**

| Term | VIF |
| --- | --- |
| sofa | 2.84 |
| cci | 2.54 |
| chf | 2.37 |
| ckd | 2.25 |
| male | 2.22 |
| apsiii | 2.20 |
| vasopressor_flag | 2.19 |
| mv_flag | 2.10 |
| age | 1.62 |
| dm | 1.61 |
| creatinine | 1.61 |
| mi | 1.52 |
| copd | 1.37 |
| malignancy | 1.35 |
| mbp_mean | 1.34 |
| stroke | 1.34 |
| hemoglobin | 1.29 |
| MVB, per point | 1.26 |
| rrt_flag | 1.26 |
| liver_disease | 1.21 |

**Supplementary Table S7. Adjusted subgroup interaction tests**

| Subgroup variable | Interaction term | Interaction HR | 95% CI | P interaction | N | Events |
| --- | --- | --- | --- | --- | --- | --- |
| SOFA ≥5 | burden x SOFA ≥5 | 0.99 | 0.99 (0.96-1.02) | 0.463 | 5320 | 1969 |
| APS III ≥50 | burden x APS III ≥50 | 0.97 | 0.97 (0.94-1.01) | 0.116 | 5320 | 1969 |
| CCI ≥6 | burden x CCI ≥6 | 1.00 | 1.00 (0.96-1.03) | 0.843 | 5320 | 1969 |
| Vasopressor use | burden x Vasopressor use | 0.95 | 0.95 (0.92-0.99) | 0.007 | 5320 | 1969 |
| Mechanical ventilation | burden x Mechanical ventilation | 0.95 | 0.95 (0.92-0.98) | 0.002 | 5320 | 1969 |

**Supplementary Table S8. Full Cox coefficients**

| Model | N | Events | Term | HR (95% CI) | P value |
| --- | --- | --- | --- | --- | --- |
| Clinical model | 5320 | 1969 | age | 1.02 (1.01-1.02) | <0.001 |
| Clinical model | 5320 | 1969 | male | 0.98 (0.90-1.08) | 0.731 |
| Clinical model | 5320 | 1969 | sofa | 1.07 (1.05-1.09) | <0.001 |
| Clinical model | 5320 | 1969 | apsiii | 1.02 (1.02-1.02) | <0.001 |
| Clinical model | 5320 | 1969 | cci | 1.20 (1.16-1.23) | <0.001 |
| Clinical model | 5320 | 1969 | mi | 1.06 (0.96-1.17) | 0.280 |
| Clinical model | 5320 | 1969 | chf | 1.08 (0.97-1.19) | 0.142 |
| Clinical model | 5320 | 1969 | stroke | 1.03 (0.91-1.17) | 0.611 |
| Clinical model | 5320 | 1969 | copd | 0.93 (0.84-1.04) | 0.189 |
| Clinical model | 5320 | 1969 | liver_disease | 1.10 (0.93-1.29) | 0.259 |
| Clinical model | 5320 | 1969 | dm | 0.79 (0.71-0.88) | <0.001 |
| Clinical model | 5320 | 1969 | ckd | 0.72 (0.64-0.82) | <0.001 |
| Clinical model | 5320 | 1969 | malignancy | 1.13 (0.98-1.32) | 0.094 |
| Clinical model | 5320 | 1969 | vasopressor_flag | 1.10 (0.99-1.23) | 0.085 |
| Clinical model | 5320 | 1969 | mv_flag | 1.12 (1.00-1.25) | 0.045 |
| Clinical model | 5320 | 1969 | rrt_flag | 1.17 (0.97-1.42) | 0.102 |
| Clinical model | 5320 | 1969 | creatinine | 0.98 (0.95-1.02) | 0.314 |
| Clinical model | 5320 | 1969 | hemoglobin | 0.98 (0.96-1.00) | 0.062 |
| Clinical model | 5320 | 1969 | mbp_mean | 1.00 (0.99-1.00) | 0.093 |
| Clinical + continuous MVB | 5320 | 1969 | age | 1.01 (1.01-1.02) | <0.001 |
| Clinical + continuous MVB | 5320 | 1969 | male | 1.01 (0.92-1.11) | 0.803 |
| Clinical + continuous MVB | 5320 | 1969 | sofa | 1.07 (1.04-1.09) | <0.001 |
| Clinical + continuous MVB | 5320 | 1969 | apsiii | 1.02 (1.02-1.02) | <0.001 |
| Clinical + continuous MVB | 5320 | 1969 | cci | 1.20 (1.16-1.23) | <0.001 |
| Clinical + continuous MVB | 5320 | 1969 | mi | 1.06 (0.96-1.17) | 0.258 |
| Clinical + continuous MVB | 5320 | 1969 | chf | 0.97 (0.87-1.07) | 0.531 |
| Clinical + continuous MVB | 5320 | 1969 | stroke | 1.03 (0.91-1.16) | 0.617 |
| Clinical + continuous MVB | 5320 | 1969 | copd | 0.92 (0.83-1.02) | 0.109 |
| Clinical + continuous MVB | 5320 | 1969 | liver_disease | 1.08 (0.92-1.27) | 0.346 |
| Clinical + continuous MVB | 5320 | 1969 | dm | 0.83 (0.74-0.92) | <0.001 |
| Clinical + continuous MVB | 5320 | 1969 | ckd | 0.71 (0.63-0.81) | <0.001 |
| Clinical + continuous MVB | 5320 | 1969 | malignancy | 1.13 (0.98-1.31) | 0.093 |
| Clinical + continuous MVB | 5320 | 1969 | vasopressor_flag | 1.09 (0.97-1.22) | 0.143 |
| Clinical + continuous MVB | 5320 | 1969 | mv_flag | 1.14 (1.02-1.27) | 0.019 |
| Clinical + continuous MVB | 5320 | 1969 | rrt_flag | 1.15 (0.96-1.40) | 0.136 |
| Clinical + continuous MVB | 5320 | 1969 | creatinine | 0.98 (0.95-1.01) | 0.272 |
| Clinical + continuous MVB | 5320 | 1969 | hemoglobin | 0.98 (0.96-1.00) | 0.060 |
| Clinical + continuous MVB | 5320 | 1969 | mbp_mean | 1.00 (0.99-1.00) | 0.175 |
| Clinical + continuous MVB | 5320 | 1969 | MVB, per point | 1.07 (1.05-1.09) | <0.001 |
| Clinical + MVB >=4 | 5320 | 1969 | age | 1.02 (1.01-1.02) | <0.001 |
| Clinical + MVB >=4 | 5320 | 1969 | male | 1.01 (0.92-1.11) | 0.840 |
| Clinical + MVB >=4 | 5320 | 1969 | sofa | 1.07 (1.05-1.09) | <0.001 |
| Clinical + MVB >=4 | 5320 | 1969 | apsiii | 1.02 (1.02-1.02) | <0.001 |
| Clinical + MVB >=4 | 5320 | 1969 | cci | 1.20 (1.16-1.23) | <0.001 |
| Clinical + MVB >=4 | 5320 | 1969 | mi | 1.07 (0.96-1.18) | 0.222 |
| Clinical + MVB >=4 | 5320 | 1969 | chf | 1.00 (0.90-1.11) | 0.988 |
| Clinical + MVB >=4 | 5320 | 1969 | stroke | 1.03 (0.91-1.16) | 0.654 |
| Clinical + MVB >=4 | 5320 | 1969 | copd | 0.92 (0.83-1.03) | 0.135 |
| Clinical + MVB >=4 | 5320 | 1969 | liver_disease | 1.08 (0.92-1.27) | 0.346 |
| Clinical + MVB >=4 | 5320 | 1969 | dm | 0.82 (0.74-0.91) | <0.001 |
| Clinical + MVB >=4 | 5320 | 1969 | ckd | 0.72 (0.63-0.81) | <0.001 |
| Clinical + MVB >=4 | 5320 | 1969 | malignancy | 1.13 (0.97-1.31) | 0.114 |
| Clinical + MVB >=4 | 5320 | 1969 | vasopressor_flag | 1.09 (0.97-1.22) | 0.138 |
| Clinical + MVB >=4 | 5320 | 1969 | mv_flag | 1.13 (1.02-1.26) | 0.025 |
| Clinical + MVB >=4 | 5320 | 1969 | rrt_flag | 1.16 (0.96-1.40) | 0.123 |
| Clinical + MVB >=4 | 5320 | 1969 | creatinine | 0.98 (0.95-1.01) | 0.201 |
| Clinical + MVB >=4 | 5320 | 1969 | hemoglobin | 0.98 (0.96-1.00) | 0.059 |
| Clinical + MVB >=4 | 5320 | 1969 | mbp_mean | 1.00 (0.99-1.00) | 0.144 |
| Clinical + MVB >=4 | 5320 | 1969 | MVB >=4 | 1.35 (1.22-1.48) | <0.001 |
| Clinical + separate TR/MR/AR grades | 5320 | 1969 | age | 1.01 (1.01-1.02) | <0.001 |
| Clinical + separate TR/MR/AR grades | 5320 | 1969 | male | 1.02 (0.93-1.11) | 0.739 |
| Clinical + separate TR/MR/AR grades | 5320 | 1969 | sofa | 1.07 (1.04-1.09) | <0.001 |
| Clinical + separate TR/MR/AR grades | 5320 | 1969 | apsiii | 1.02 (1.02-1.02) | <0.001 |
| Clinical + separate TR/MR/AR grades | 5320 | 1969 | cci | 1.20 (1.16-1.23) | <0.001 |
| Clinical + separate TR/MR/AR grades | 5320 | 1969 | mi | 1.06 (0.96-1.18) | 0.235 |
| Clinical + separate TR/MR/AR grades | 5320 | 1969 | chf | 0.96 (0.87-1.07) | 0.501 |
| Clinical + separate TR/MR/AR grades | 5320 | 1969 | stroke | 1.03 (0.92-1.17) | 0.590 |
| Clinical + separate TR/MR/AR grades | 5320 | 1969 | copd | 0.92 (0.83-1.02) | 0.101 |
| Clinical + separate TR/MR/AR grades | 5320 | 1969 | liver_disease | 1.07 (0.91-1.26) | 0.407 |
| Clinical + separate TR/MR/AR grades | 5320 | 1969 | dm | 0.82 (0.74-0.92) | <0.001 |
| Clinical + separate TR/MR/AR grades | 5320 | 1969 | ckd | 0.71 (0.63-0.81) | <0.001 |
| Clinical + separate TR/MR/AR grades | 5320 | 1969 | malignancy | 1.14 (0.98-1.32) | 0.088 |
| Clinical + separate TR/MR/AR grades | 5320 | 1969 | vasopressor_flag | 1.08 (0.97-1.21) | 0.164 |
| Clinical + separate TR/MR/AR grades | 5320 | 1969 | mv_flag | 1.14 (1.03-1.28) | 0.016 |
| Clinical + separate TR/MR/AR grades | 5320 | 1969 | rrt_flag | 1.15 (0.95-1.39) | 0.143 |
| Clinical + separate TR/MR/AR grades | 5320 | 1969 | creatinine | 0.98 (0.95-1.01) | 0.267 |
| Clinical + separate TR/MR/AR grades | 5320 | 1969 | hemoglobin | 0.98 (0.96-1.00) | 0.072 |
| Clinical + separate TR/MR/AR grades | 5320 | 1969 | mbp_mean | 1.00 (0.99-1.00) | 0.142 |
| Clinical + separate TR/MR/AR grades | 5320 | 1969 | TR grade, per point | 1.09 (1.05-1.14) | <0.001 |
| Clinical + separate TR/MR/AR grades | 5320 | 1969 | MR grade, per point | 1.06 (1.02-1.11) | 0.002 |

Supplementary Table S9. Proportional hazards diagnostics for primary and individual-valve models

| Model | Term | test_statistic | P value | -log2(p) |
| --- | --- | --- | --- | --- |
| Clinical + continuous MVB | age | 0.57 | 0.450 | 1.15 |
| Clinical + continuous MVB | apsiii | 20.34 | <0.001 | 17.24 |
| Clinical + continuous MVB | MVB, per point | 4.61 | 0.032 | 4.97 |
| Clinical + continuous MVB | cci | 0.70 | 0.404 | 1.31 |
| Clinical + continuous MVB | chf | 28.61 | <0.001 | 23.43 |
| Clinical + continuous MVB | ckd | 0.15 | 0.702 | 0.51 |
| Clinical + continuous MVB | copd | 0.14 | 0.709 | 0.50 |
| Clinical + continuous MVB | creatinine | 0.62 | 0.430 | 1.22 |
| Clinical + continuous MVB | dm | 0.55 | 0.457 | 1.13 |
| Clinical + continuous MVB | hemoglobin | 6.65 | 0.010 | 6.66 |
| Clinical + continuous MVB | liver_disease | 0.43 | 0.511 | 0.97 |
| Clinical + continuous MVB | male | 3.54 | 0.060 | 4.06 |
| Clinical + continuous MVB | malignancy | 5.69 | 0.017 | 5.87 |
| Clinical + continuous MVB | mbp_mean | 0.74 | 0.391 | 1.35 |
| Clinical + continuous MVB | mi | 9.86 | 0.002 | 9.21 |
| Clinical + continuous MVB | mv_flag | 21.10 | <0.001 | 17.81 |
| Clinical + continuous MVB | rrt_flag | 0.84 | 0.360 | 1.47 |
| Clinical + continuous MVB | sofa | 1.03 | 0.310 | 1.69 |
| Clinical + continuous MVB | stroke | 1.86 | 0.173 | 2.53 |
| Clinical + continuous MVB | vasopressor_flag | 6.70 | 0.010 | 6.70 |
| Clinical + separate TR/MR/AR grades | age | 0.62 | 0.433 | 1.21 |
| Clinical + separate TR/MR/AR grades | apsiii | 20.21 | <0.001 | 17.14 |
| Clinical + separate TR/MR/AR grades | AR grade, per point | 0.00 | 0.969 | 0.05 |
| Clinical + separate TR/MR/AR grades | cci | 0.61 | 0.433 | 1.21 |
| Clinical + separate TR/MR/AR grades | chf | 29.40 | <0.001 | 24.02 |
| Clinical + separate TR/MR/AR grades | ckd | 0.18 | 0.670 | 0.58 |
| Clinical + separate TR/MR/AR grades | copd | 0.14 | 0.706 | 0.50 |
| Clinical + separate TR/MR/AR grades | creatinine | 0.72 | 0.397 | 1.33 |
| Clinical + separate TR/MR/AR grades | dm | 0.62 | 0.431 | 1.21 |
| Clinical + separate TR/MR/AR grades | hemoglobin | 7.01 | 0.008 | 6.95 |
| Clinical + separate TR/MR/AR grades | liver_disease | 0.44 | 0.506 | 0.98 |
| Clinical + separate TR/MR/AR grades | male | 3.40 | 0.065 | 3.94 |
| Clinical + separate TR/MR/AR grades | malignancy | 5.80 | 0.016 | 5.97 |
| Clinical + separate TR/MR/AR grades | mbp_mean | 0.84 | 0.360 | 1.47 |
| Clinical + separate TR/MR/AR grades | mi | 8.82 | 0.003 | 8.39 |
| Clinical + separate TR/MR/AR grades | MR grade, per point | 4.19 | 0.041 | 4.62 |
| Clinical + separate TR/MR/AR grades | mv_flag | 20.96 | <0.001 | 17.70 |
| Clinical + separate TR/MR/AR grades | rrt_flag | 0.81 | 0.367 | 1.45 |
| Clinical + separate TR/MR/AR grades | sofa | 1.12 | 0.289 | 1.79 |
| Clinical + separate TR/MR/AR grades | stroke | 1.82 | 0.177 | 2.49 |
| Clinical + separate TR/MR/AR grades | TR grade, per point | 0.34 | 0.560 | 0.84 |
| Clinical + separate TR/MR/AR grades | vasopressor_flag | 7.02 | 0.008 | 6.95 |

P values below 0.05 indicate departures from the proportional hazards assumption. In the primary model, continuous MVB showed a small departure from proportionality (P = 0.032); therefore, its Cox estimate is interpreted as an average association across the 365-day follow-up rather than a strictly time-invariant effect.

Supplementary Table S9A. Proportional hazards diagnostics for revised sensitivity models

| Model | Diagnostic | P value |
| --- | --- | --- |
| AS/MS missing-aware cleaned model | Global Schoenfeld test (chi-square 117.390; df=24) | <0.001 |
| AS/MS missing-aware cleaned model | MVB, per point | 0.036 |
| AS/MS missing-aware cleaned model | Cleaned AS proxy positive | 0.997 |
| AS/MS missing-aware cleaned model | AS source unavailable | 0.068 |
| AS/MS missing-aware cleaned model | MS proxy positive | 0.439 |
| AS/MS missing-aware cleaned model | MS source unavailable | 0.152 |
| Strict RV/LVEF model | Global Schoenfeld test (chi-square 92.485; df=24) | <0.001 |
| Strict RV/LVEF model | MVB, per point | 0.056 |
| Strict RV/LVEF model | LVEF, per 1% | 0.153 |
| Strict RV/LVEF model | Explicit RV dysfunction | 0.543 |
| Strict RV/LVEF model | RV function indeterminate | 0.806 |
| Strict RV/LVEF model | RV-function text unavailable | 0.407 |

Note: Global proportional-hazards tests were significant in both revised sensitivity models. The MVB term showed a small departure in the AS/MS model (P = 0.036) and did not cross the conventional 0.05 threshold in the strict RV/LVEF model (P = 0.056).

**Supplementary Table S10. Restricted cubic spline analysis of MVB and one-year mortality.**

| Analysis | Exposure | Outcome | P non-linearity | Interpretation |
| --- | --- | --- | --- | --- |
| Restricted cubic spline with three knots | Continuous MVB | 1-year all-cause mortality | 0.43 | No material non-linearity detected |

Supplementary Figure S1. Adjusted associations of MVB, individual valve grades, and revised sensitivity models with one-year all-cause mortality. The AS/MS sensitivity estimate uses the cleaned, missing-aware model, and the LVEF/RV estimate uses the strict structured RV-function classification.

Note: MVB is the exact sum of TR, MR, and AR component grades. Therefore, unpenalized models containing MVB and all three component grades are structurally collinear; these models are not interpreted as primary inference.
